# The CelB Gene Mediates Erythritol-Induced Inhibition of Exopolysaccharide Synthesis in *Streptococcus mutans*

**DOI:** 10.3390/microorganisms14040782

**Published:** 2026-03-30

**Authors:** Yang Chen, Yuwei Gu, Junxin Zhu, Dongmei Wang, Yixiang Wang

**Affiliations:** 1 Department of Pediatric Dentistry, Peking University School and Hospital of Stomatology, Beijing 100081, China; drchenyang1994@163.com (Y.C.);; 2 National Center for Stomatology, Peking University School and Hospital of Stomatology, Beijing 100081, China; 3 National Clinical Research Center for Oral Diseases, Peking University School and Hospital of Stomatology, Beijing 100081, China; 4 National Engineering Research Center of Oral Biomaterials and Digital Medical Devices, Peking University School and Hospital of Stomatology, Beijing 100081, China; 5 Beijing Key Laboratory of Digital Stomatology, Peking University School and Hospital of Stomatology, Beijing 100081, China; 6 NHC Key Laboratory of Digital Stomatology, Peking University School and Hospital of Stomatology, Beijing 100081, China; 7 The Second Clinical Division, Peking University School and Hospital of Stomatology, Beijing 100081, China; 8 Central Laboratory, Peking University School and Hospital of Stomatology, Beijing 100081, China

**Keywords:** dental caries, *Streptococcus mutans*, erythritol, biofilm, *celB*, EPS

## Abstract

*Streptococcus mutans* is a primary contributor to dental caries due to its ability to form biofilms rich in extracellular polysaccharides (EPS). While erythritol has been recognized for its anti-cariogenic effects, the molecular pathways involved have remained unclear. In this study, we combined phenotypic and transcriptomic approaches to uncover the mechanism by which erythritol inhibits EPS synthesis. We found that erythritol treatment significantly reduces EPS production and biofilm density, and that these changes are accompanied by marked downregulation of the phosphotransferase system (PTS), particularly the *celB* gene. Functional validation through gene deletion demonstrated that loss of *celB* mimics the effects of erythritol, resulting in reduced bacterial growth, impaired biofilm formation and decreased EPS production. Our results establish *celB* as a key mediator of erythritol-induced biofilm inhibition and suggest that targeting carbohydrate transport systems could offer a novel approach to caries prevention.

## 1. Introduction

Dental caries is one of the most prevalent biofilm-associated infectious diseases worldwide. Among oral pathogens, *Streptococcus mutans* (*S. mutans*) is widely recognized as a major etiological agent due to its capacity to adhere to tooth surfaces, produce acid from dietary carbohydrates, and synthesize extracellular polysaccharides (EPS) that constitute the structural matrix of dental biofilms [1]. EPS, primarily generated by glucosyltransferases (Gtfs), facilitates bacterial accumulation, stabilizes biofilm architecture, and limits the penetration of antimicrobial agents [2]. Consequently, interference with EPS synthesis has emerged as a promising strategy for caries control.

Erythritol, a four-carbon sugar alcohol extensively used as a non-caloric sweetener, has gained attention for its potential anti-cariogenic properties. Unlike fermentable sugars, erythritol is not metabolized by *S. mutans* to produce organic acids [3]. In addition, several in vitro and clinical studies have reported that erythritol reduces biofilm accumulation and decreases insoluble polysaccharide formation [4,5]. However, most studies have focused on phenotypic outcomes or changes in glucosyltransferase gene expression, and the upstream regulatory pathways involved in erythritol-mediated biofilm modulation remain poorly characterized.

In *S. mutans*, carbohydrate uptake and sensing are largely mediated by the phosphoenolpyruvate-dependent phosphotransferase system (PTS) [6]. Beyond sugar transport, the PTS participates in carbon catabolite repression and coordinates metabolic status with virulence-associated gene expression [6,7,8]. The cellobiose-specific PTS, encoded by the *cel* operon, includes the *celB* gene, which encodes the IIB component of the transport complex [9]. Although the *cel* operon has been primarily studied in the context of carbohydrate transport and is known to be subject to transcriptional regulation [10]; its potential contribution to EPS production and biofilm organization has not been clearly defined.

Given the increasing use of non-fermentable sugar substitutes in caries prevention, it is important to determine whether erythritol exerts solely passive effects or actively modulates bacterial virulence pathways. In particular, EPS synthesis plays a central role in plaque stability, bacterial adhesion, and resistance to antimicrobial agents, making it a clinically relevant target. Therefore, elucidating the molecular mechanisms by which erythritol influences EPS production in *S. mutans* is of both biological and clinical significance.

## 2. Materials & Methods

### 2.1. Bacterial Strains and Culture Conditions

*Streptococcus mutans* UA159 (ATCC 700610) was used as the wild-type (WT) strain throughout this study. Bacteria were routinely cultured in brain heart infusion (BHI; Hopebio, Qingdao, China) broth at 37 °C under 5% CO_2_. Overnight cultures were diluted 1:100 into fresh BHI and grown to an optical density at 600 nm (OD_600_) of approximately 0.5. This suspension was then further diluted 1:100 into fresh medium for all subsequent experiments to ensure standardized initial inoculum and comparable physiological status across assays. When indicated, erythritol (Sigma-Aldrich, St. Louis, MO, USA) was supplemented to the medium at a final concentration of 10% (*w*/*v*), based on preliminary experiments.

### 2.2. Construction of the ΔcelB Mutant

The *celB* gene (locus tag SMU_RS07235) deletion mutant was generated using a two-step homologous recombination method as previously described [11]. Briefly, approximately 1 kb upstream and downstream flanking regions of *celB* were amplified by PCR from the *S. mutans* UA159 genome. These fragments were fused with an erythromycin resistance cassette (*erm*) by overlapping PCR. The resulting linear construct was transformed into competent *S. mutans* UA159 cells as described previously. Transformants were selected on BHI agar plates containing 10 μg/mL erythromycin. The deletion of *celB* was confirmed by colony PCR and further verified by quantitative real-time PCR (qPCR) to ensure the absence of *celB* transcript. All primers used in this study are listed in Supplementary Table S1.

### 2.3. Growth Curve Analysis

Overnight cultures of *Streptococcus mutans* UA159 were diluted 1:100 into fresh BHI with or without 10% erythritol in sterile 96-well microplates. Similarly, the growth of wild type (WT) and Δ*celB* strains was compared in BHI without erythritol. The plates were incubated at 37 °C in a microplate reader (BioTek Instruments, Winooski, VT, USA). The OD_600_ was measured every hour for 12 h, with brief shaking before each reading. 

### 2.4. Biofilm Formation and EPS Quantification

Biofilm formation was assessed using the crystal violet staining method as previously described [12]. Briefly, overnight cultures of *S. mutans* were adjusted to 1 × 10^7^ CFU/mL in fresh BHI with or without 10% erythritol. Two-milliliter aliquots of the bacterial suspension were inoculated into 24-well plates (Biofil, Guangzhou, China) and incubated statically at 37 °C for 12 h. After incubation, the culture medium was gently aspirated, and the wells were washed twice with phosphate-buffered saline (PBS) to remove loosely adherent cells.

For biomass formation assessment, biofilms were fixed with 1 mL of methanol for 15 min, air-dried, and stained with 1 mL of 0.1% (*w*/*v*) crystal violet for 20 min. Excess stain was removed by washing with running water. The bound crystal violet was solubilized with 1 mL of 33% (*v*/*v*) acetic acid, and the absorbance was measured at 575 nm (OD_575_) using a microplate reader (BioTek Instruments, Winooski, VT, USA). Blank wells containing medium only were included as negative controls in the crystal violet assay.

For EPS quantification, water-insoluble EPS was measured using the anthrone-sulfuric acid method with modifications [13]. After biofilm formation in 24-well plates as described above, the biofilms were gently washed with PBS, scraped, and collected in 1 mL of deionized water. Samples were centrifuged at 10,000× *g* for 10 min at 4 °C, and the supernatant was discarded. The pellet was washed twice with PBS and then resuspended in 1 mL of 1 M NaOH. After incubation at 37 °C for 2 h to extract water-insoluble glucans, 200 μL of the alkali-soluble carbohydrate solution was mixed with anthrone-sulfuric acid reagent and heated at 95 °C for 5 min. The absorbance was measured at 625 nm using a microplate reader (BioTek Instruments, Winooski, VT, USA).

### 2.5. Biofilm Architecture Analysis by SEM and CLSM

For both SEM and CLSM, *S. mutans* biofilms were cultured on sterile glass coverslips in 24-well plates as described in Section 2.4. After 12 h incubation, coverslips were gently washed with PBS and processed accordingly.

For SEM, biofilms were fixed with 2.5% glutaraldehyde overnight at 4 °C, post-fixed with 1% osmium tetroxide for 1 h, dehydrated through a graded ethanol series, critical-point dried, sputter-coated with gold-palladium, and observed under a scanning electron microscope (SU8000, Hitachi, Tokyo, Japan) at 10.0 kV.

For CLSM, dual fluorescent labeling was performed to visualize bacteria and EPS. Biofilms were incubated with rabbit anti-FtsZ antibody (1:200) at 37 °C for 60 min, washed, then incubated with Cy3-conjugated secondary antibody (1:500) at 37 °C for 60 min in the dark. After washing, samples were stained with FITC-ConA (1:200) at 37 °C for 60 min in the dark to label EPS. Coverslips were washed and placed on glass slides for immediate observation. Images were acquired using a Nikon confocal microscope. Cy3 fluorescence (FtsZ) was visualized at 570–620 nm, and FITC fluorescence (EPS) at 500–550 nm. At least three random fields were scanned per sample. Three-dimensional reconstructions and quantitative analysis were performed using COMSTAT software. Biofilm structural parameters, including biomass (as calculated by COMSTAT, μm^3^/μm^2^), were quantified.

### 2.6. RNA Extraction, Sequencing and Bioinformatics Analysis

*S. mutans* UA159 was cultured in BHI with or without 10% erythritol for 12 h. Total RNA was extracted by Novogene Corporation (Beijing, China) following their standard protocols. RNA quality was assessed using an Agilent 2100 Bioanalyzer (Agilent Technologies, Inc., Santa Clara, CA, USA). Strand-specific cDNA libraries were constructed using the dUTP method. Library quality was evaluated using a Qubit 2.0 Fluorometer and an Agilent 2100 Bioanalyzer, and the effective concentration was quantified by qPCR. Qualified libraries were sequenced on an Illumina platform with paired-end reads.

Raw reads were processed using fastp to obtain clean reads by removing adapter-contaminated, poly-N-containing, and low-quality reads. Clean reads were aligned to the *S. mutans* UA159 reference genome (NC_004350) using HISAT2.

Differential expression analysis between control and erythritol-treated groups was performed using DESeq2 (version 1.42.0). Genes with an adjusted *p*-value ≤ 0.05 were considered significantly differentially expressed. GO and KEGG enrichment analyses were performed using clusterProfiler (version 4.8.1). The gene expression matrix is available as Supplementary Table S2.

### 2.7. Quantitative Real-Time PCR

Total RNA was extracted from *S. mutans* cells using TRIzol reagent (Invitrogen, Carlsbad, CA, USA) according to the manufacturer’s instructions. RNA quality and concentration were assessed by agarose gel electrophoresis and spectrophotometry (NanoDrop, Thermo Fisher Scientific, Waltham, MA, USA). First-strand cDNA was synthesized from 1 μg of total RNA using the 1-Step RT kit (Abclonal, Wuhan, China) following the manufacturer’s protocol. Quantitative PCR was performed in a QuantStudio 6 Real-Time PCR System (Applied Biosystems, Foster City, CA, USA) using SYBR Green Master Mix (Applied Biosystems). The 16S rRNA gene was used as an endogenous control for normalization. Relative expression levels of target genes were calculated using the 2^−ΔΔct^ method. All reactions were performed in triplicate with three independent biological replicates. Primer sequences are listed in Supplementary Table S1.

### 2.8. Statistical Analysis

All assays were performed in three independent experiments, each containing technical triplicates. Data are presented as the mean ± standard deviation (SD). Due to the small sample size per group (*n* = 3), reliable assessment of normality and homogeneity of variances was not feasible. Considering the robustness of Student’s *t*-test under small sample conditions and common practices in the field, comparisons between two groups were analyzed using an unpaired two-tailed Student’s *t*-test in this study. Statistical analyses were performed using GraphPad Prism version 9.0 (GraphPad Software, San Diego, CA, USA). A *p*-value of less than 0.05 was considered statistically significant.

## 3. Results

### 3.1. Erythritol Inhibits EPS Production and Alters Biofilm Architecture in S. mutans

**Figure 1:**
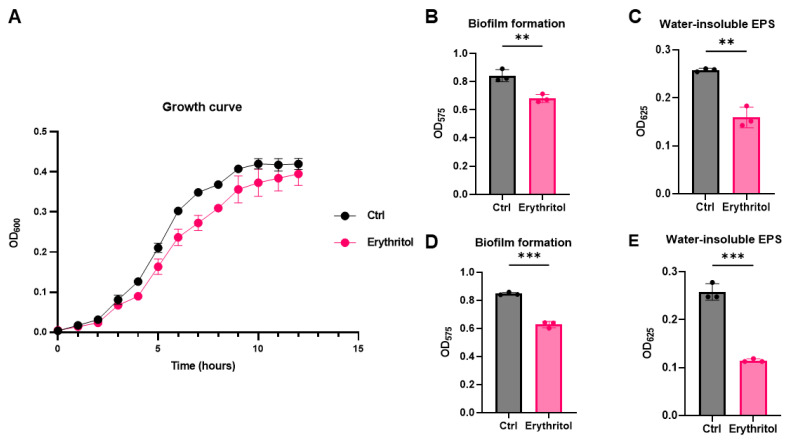
Erythritol reduces growth, biofilm formation, and EPS production in *Streptococcus mutans*. (**A**) Growth curves of *S. mutans* cultured in BHI with or without 10% erythritol under aerobic condition. OD_600_ was monitored for 12 h. (**B**) Quantification of biofilm formation under aerobic condition at 12 h using crystal violet staining. (**C**) Quantification of water-insoluble EPS by anthrone–sulfuric acid assay under aerobic conditions. (**D**) Quantification of biofilm under anaerobic condition. (**E**) Quantification of water-insoluble EPS by anthrone–sulfuric acid assay under anaerobic condition. Data are presented as mean ± SD (*n* = 3). Statistical significance was determined using an unpaired two-tailed Student’s *t*-test. (**: *p* < 0.01, ***: *p* < 0.001).

To investigate the effect of erythritol on *S. mutans*, we first examined bacterial growth in the presence of 10% erythritol under aerobic conditions. Growth curve analysis showed that erythritol delayed the growth of *S. mutans* during the exponential phase (Figure 1A). Crystal violet staining revealed a significant difference in biofilm formation was observed between groups (Figure 1B). In addition, when EPS production was quantified by the anthrone-sulfuric acid method, erythritol treatment significantly reduced EPS levels compared to the control (Figure 1C). To further evaluate whether these effects persist under conditions more representative of the oral environment, additional experiments were performed under anaerobic conditions. Consistent with the results observed under aerobic conditions, erythritol significantly reduced biofilm formation and EPS production (Figure 1D,E), indicating that its inhibitory effects are maintained in low-oxygen environments.

**Figure 2:**
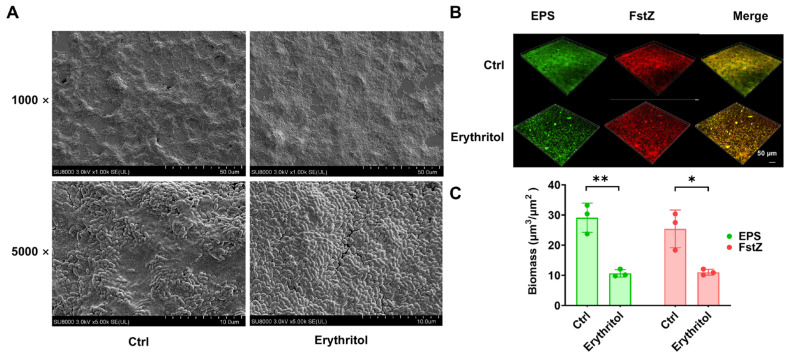
Erythritol alters biofilm architecture and reduces EPS in *S. mutans.* (**A**) Representative SEM images of *S. mutans* biofilms cultured with or without 10% erythritol for 12 h. Control biofilms showed dense structure with abundant extracellular matrix; erythritol-treated biofilms appeared sparse with markedly reduced matrix. (**B**) Representative CLSM images showing bacterial cells labeled with anti-FtsZ antibody/Cy3 (red) and EPS labeled with FITC-ConA (green) in control and erythritol-treated biofilms. Yellow color shows the merged signals by red and green. (**C**) Quantification of CLSM data showing relative fluorescence intensity of bacterial cells (Cy3) and EPS (FITC) signals. Data are presented as mean ± SD (*n* = 3 fields per group). Statistical significance was determined using an unpaired two-tailed Student’s *t*-test. (*: *p* < 0.05, **: *p* < 0.01).

SEM imaging revealed that control biofilms were dense and covered with abundant extracellular matrix, while erythritol-treated biofilms appeared sparser with markedly reduced matrix (Figure 2A). CLSM further confirmed these observations, showing that both bacterial cells (FtsZ, red) and EPS (FITC-conA, green) were visibly decreased in the erythritol-treated group (Figure 2B,C). These results indicate that erythritol specifically inhibits EPS synthesis without affecting total biofilm formation at 12 h.

### 3.2. Transcriptomic Analysis Reveals Metabolic Remodeling in S. mutans upon Erythritol Treatment

**Figure 3:**
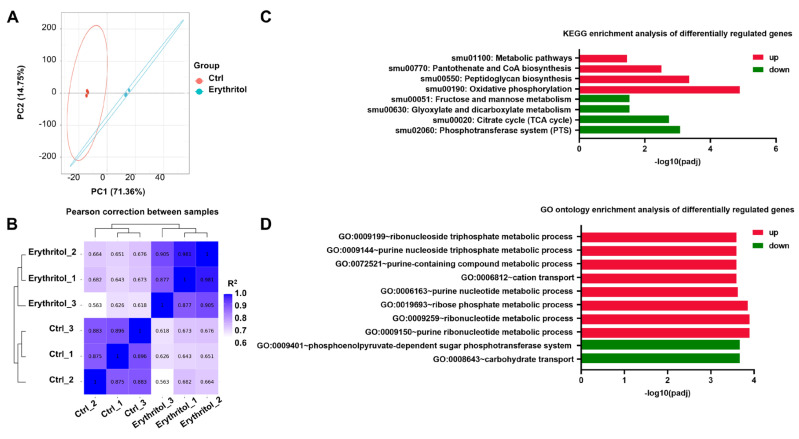
Transcriptomic analysis reveals metabolic remodeling in *S. mutans* upon erythritol treatment. (**A**) Principal component analysis (PCA) of RNA-seq data from *S. mutans* cultured with or without 10% erythritol (*n* = 3). Each dot represents one replicate; ellipses indicate 95% confidence intervals. (**B**) Pearson correlation heatmap showing intra- and inter-group sample correlations. Color scale represents *R*^2^ values. (**C**) KEGG pathway enrichment of significantly differentially expressed genes (|log_2_ fold change| ≥ 1, adjusted *p* < 0.05). The *x*-axis represents −log_10_ (adjusted *p*-value). Red bars indicate enrichment among upregulated genes, and green bars indicate enrichment among downregulated genes. (**D**) GO enrichment analysis of differentially expressed genes. The *x*-axis represents −log_10_ (adjusted *p*-value).

**Figure 4:**
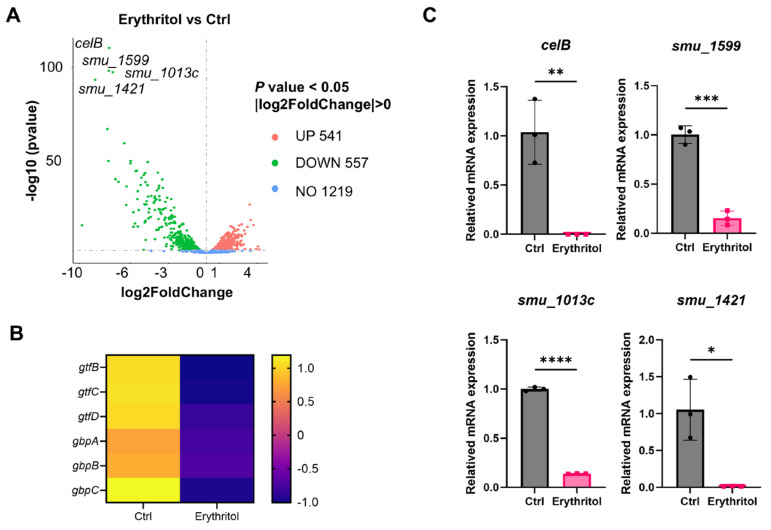
*celB* is the most significantly downregulated gene upon erythritol treatment. (**A**) Volcano plot of differentially expressed genes in *S. mutans* treated with 10% erythritol versus control. The *x*-axis represents log_2_ fold change; the *y*-axis represents −log_10_ adjusted *p*-value. Significantly downregulated genes (|log_2_FC| ≥ 1, padj < 0.05) are highlighted in blue, and upregulated genes in red. Key downregulated genes including *celB*, *smu_1599*, *smu_1031c*, and *smu_1421* are labeled. (**B**) The heatmap displays normalized expression patterns of three glucosyltransferase genes (*gtfB*, *gtfC*, *gtfD*) and three glucan-binding protein genes (*gbpA*, *gbpB*, *gbpC*) under control and erythritol treatment conditions (*n* = 3). (**C**) Quantitative real-time PCR (qPCR) validation of *celB* expression in *S. mutans* treated with or without 10% erythritol. Expression was normalized to 16S rRNA. Data are presented as mean ± SD (*n* = 3). (*: *p* < 0.05, **: *p* < 0.01, ***: *p* < 0.001, ****: *p* < 0.0001).

To elucidate the mechanism underlying erythritol-mediated EPS inhibition, we performed transcriptomic analysis of *S. mutans* cultured with or without 10% erythritol. PCA showed clear separation between control and erythritol-treated groups along PC1 (71.36% variance), with samples clustering tightly within groups (Figure 3A). Pearson correlation analysis confirmed high intra-group (R^2^ ≈ 0.9–1.0) and low inter-group correlations (R^2^ ≈ 0.56–0.68) (Figure 3B), validating data reproducibility. KEGG enrichment analysis revealed that genes involved in the phosphotransferase system (PTS) were the most significantly downregulated, along with those in the TCA cycle and fructose/mannose metabolism (Figure 3C). In contrast, oxidative phosphorylation and peptidoglycan biosynthesis pathways were upregulated. GO enrichment showed consistent trends: downregulated processes were enriched in carbohydrate transport and PTS, while upregulated processes included nucleotide metabolism and cation transport (Figure 3D). These results indicate that erythritol suppresses sugar uptake and central carbon metabolism while upregulating energy-generating pathways. Among the differentially expressed genes, four were markedly downregulated upon erythritol treatment, including *celB* (encoding a PTS system IIB component), *smu_1599*, *smu_1031c*, and *smu_1421*. The volcano plot highlighted these as the most substantially downregulated genes (Figure 4A). The heatmap demonstrated that the glucosyltransferase (gtf) and glucan-binding protein (gbp) genes strongly downregulated in the erythritol-treated group (Figure 4B). To validate the transcriptomic results, we performed quantitative real-time PCR (qPCR) analysis. The expression level of *celB* was significantly reduced in the erythritol-treated group compared to the control, consistent with the RNA-seq data (Figure 4C). This confirmed that erythritol downregulates *celB* expression at the transcriptional level. The downregulation of PTS genes, including the *cel* operon, prompted us to investigate the role of *celB* in EPS inhibition.

### 3.3. Deletion of celB Phenocopies Erythritol Treatment and Reduces Biofilm Formation in S. mutans

**Figure 5:**
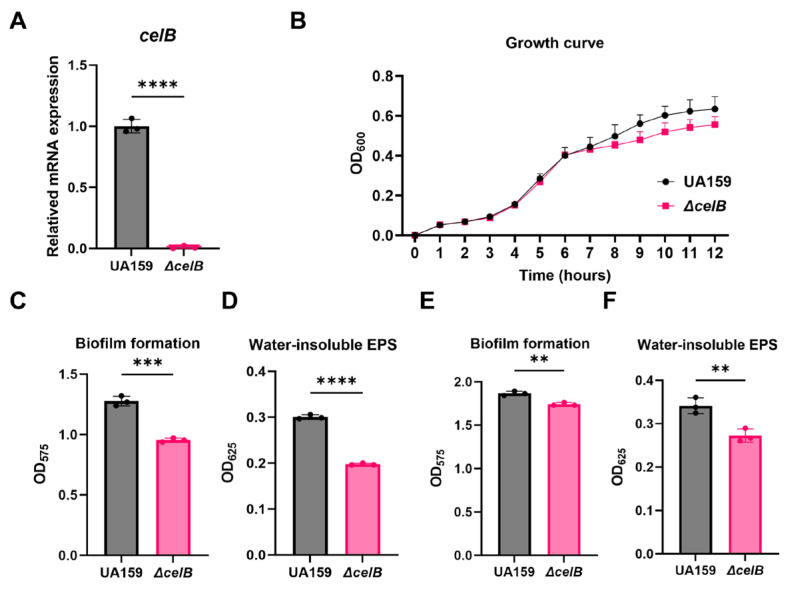
Deletion of *celB* impairs growth, biofilm formation, and EPS production in *S. mutans*. (**A**) qPCR confirmation of *celB* deletion in the Δ*celB* mutant compared to wild-type (UA159). Expression normalized to 16S rRNA. Data are presented as mean ± SD (*n* = 3). *p* < 0.001 versus UA159. (**B**) Growth curves of UA159 and Δ*celB* strains in BHI broth. OD_600_ was monitored every hour for 12 h. Data are presented as mean ± SD (*n* = 3). (**C**) Quantification of biofilm formation in UA159 and Δ*celB* strains using crystal violet staining at 12 h under aerobic conditions. (**D**) Quantification of water-insoluble EPS by anthrone-sulfuric acid assay in UA159 and Δ*celB* strains under aerobic conditions. (**E**) Quantification of biofilm under anaerobic conditions. (**F**) Quantification of water-insoluble EPS by anthrone–sulfuric acid assay under anaerobic conditions. Data are presented as mean ± SD (*n* = 3). (** *p* < 0.01, ***: *p* < 0.001, ****: *p* < 0.0001).

To investigate the functional role of *celB* in biofilm formation and EPS production, we constructed a *celB* deletion mutant (Δ*celB*) and examined its phenotypic characteristics. Quantitative real-time PCR confirmed that *celB* expression was nearly abolished in the Δ*celB* strain compared to the wild-type UA159 control (Figure 5A), confirming successful gene deletion.

Growth curve analysis showed that deletion of *celB* significantly impaired the growth of *S. mutans* (Figure 5B), indicating that *celB* is important for normal bacterial proliferation.

**Figure 6:**
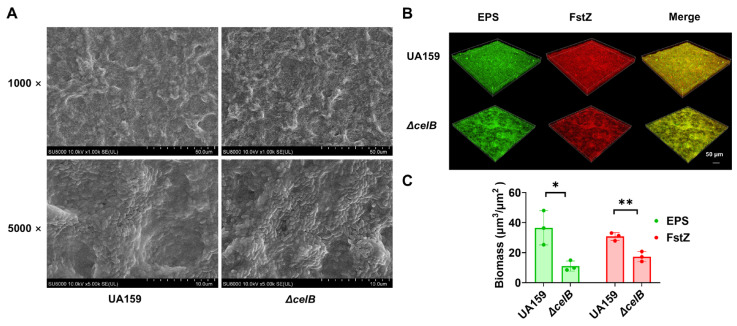
The Δ*celB* mutant displays altered biofilm architecture with reduced extracellular matrix. (**A**) Representative SEM images of biofilms formed by UA159 and Δ*celB* strains after 12 h incubation. UA159 biofilms showed dense structure embedded in abundant extracellular matrix; Δ*celB* biofilms were sparse with markedly reduced matrix. (**B**) Representative CLSM images of UA159 and Δ*celB* biofilms. Bacterial cells were labeled with anti-FtsZ antibody/Cy3 (red) and EPS was labeled with FITC-ConA (green). Yellow color shows the merged signals by red and green. (**C**) Quantification of CLSM fluorescence intensity for bacterial cells (Cy3) and EPS (FITC) in UA159 and Δ*celB* biofilms. Data are presented as mean ± SD (*n* = 3 fields per group). (*: *p* < 0.05, ** *p* < 0.01).

We next assessed biofilm formation using crystal violet staining. The Δ*celB* strain exhibited a significant reduction in biofilm formation compared to the wild-type UA159 strain (Figure 5C). Consistently, EPS production, quantified by the anthrone-sulfuric acid method, was markedly decreased in the Δ*celB* mutant (Figure 5D). Similarly, under anaerobic conditions, the Δ*celB* mutant exhibited significantly reduced biofilm formation and EPS production compared to the wild-type strain (Figure 5E,F). These findings further support the role of *celB* in regulating biofilm-associated phenotypes across different environmental conditions. SEM imaging revealed that the Δ*celB* strain formed sparse biofilms with substantially reduced extracellular matrix, whereas wild-type UA159 biofilms were dense and embedded in abundant matrix (Figure 6A). CLSM further confirmed these observations: both bacterial cells and EPS were visibly diminished in the Δ*celB* biofilms compared to wild-type UA159 (Figure 6B,C).

These results demonstrate that *celB* is required for normal biofilm formation and EPS production in *S. mutans*. The phenotypic changes observed upon *celB* deletion closely resemble those induced by erythritol treatment, supporting the notion that erythritol inhibits EPS synthesis through downregulation of *celB*.

## 4. Discussion

In this study, we demonstrate that erythritol significantly suppresses EPS production and alters biofilm architecture in *S. mutans*. Transcriptomic analysis revealed that *celB*, encoding the IIB component of the cellobiose-specific phosphotransferase system (PTS), was markedly downregulated following erythritol exposure. Functional validation showed that deletion of *celB* reduced EPS synthesis and partially reproduced the erythritol-induced phenotype. These findings suggest that *celB* contributes to matrix-associated virulence traits and may participate in the response of *S. mutans* to erythritol.

Erythritol has been widely investigated as a non-fermentable sugar alcohol with anti-cariogenic properties [5,14]. Because it is not metabolized to organic acids by *S. mutans*, erythritol does not directly contribute to enamel demineralization [15,16]. In vitro studies have consistently reported reduced biofilm formation and decreased insoluble glucan accumulation under erythritol treatment [17]. For example, previous work has demonstrated that erythritol inhibits *S. mutans* biofilm formation and reduces extracellular polysaccharide synthesis in a dose-dependent manner [18]. Clinical studies have associated erythritol consumption with lower plaque scores and reduced caries development [5]. More recently, ecological investigations have suggested that erythritol may also influence the overall structure of oral microbial communities, promoting metabolic balance within biofilms [19]. Previous *in vitro* studies investigating the effects of erythritol on *S. mutans* growth and biofilm formation have reported dose-dependent inhibitory effects at a range of concentrations [15]. In this study, we utilized a 10% erythritol concentration, which is similar to the effective doses applied in earlier investigation [3,15,17]. This concentration has been shown to effectively reduce bacterial growth and biofilm formation, allowing us to observe a robust phenotype. However, the molecular pathways linking erythritol exposure to suppression of EPS synthesis remain insufficiently defined. Importantly, the present findings have clinical implications. EPS is a key determinant of biofilm structural integrity and contributes to bacterial adhesion, acid retention, and reduced susceptibility to antimicrobial agents. Therefore, suppression of EPS synthesis represents a clinically relevant strategy for disrupting cariogenic biofilms. While erythritol is generally regarded as a non-fermentable sugar substitute, our data suggest that it may actively modulate virulence-associated pathways by influencing carbohydrate transport systems. This expands the conventional view of erythritol as a passive agent and indicates that it can contribute to caries prevention through regulatory effects on bacterial physiology. Although the concentration used in this study is higher than that typically achieved through dietary intake, localized delivery methods such as chewing gums, lozenges, or oral care formulations may transiently reach higher concentrations in the oral cavity. Moreover, even moderate modulation of carbohydrate transport systems influences the ecological balance of oral biofilms over time, potentially reducing cariogenicity. Collectively, our findings extend existing knowledge by identifying a specific carbohydrate transport component potentially involved in mediating erythritol-associated inhibition of matrix production in *S. mutans*.

In Gram-positive bacteria, the phosphoenolpyruvate-dependent PTS functions not only in carbohydrate uptake but also in coordinating metabolic signaling and global gene regulation [20]. In *S. mutans*, several sugar-specific PTS components have been implicated in biofilm formation, stress tolerance, and virulence-associated gene expression, reflecting the integration of carbohydrate sensing with pathogenic traits [21,22]. The cellobiose-specific PTS, including *celB*, has been characterized primarily in the context of carbohydrate utilization. However, its contribution to EPS biosynthesis has not been clearly established. Our data suggest that *celB* activity influences matrix production, thereby linking a carbohydrate transport component to biofilm structural regulation.

In addition to *S. mutans*, cellobiose-associated PTS systems have been implicated in biofilm-related or niche-adaptive phenotypes in other bacteria. In *Enterococcus faecalis*, genes involved in β-1,6-oligosaccharide utilization contribute to carbohydrate fitness and environmental persistence [10]. In *Streptococcus* species, carbohydrate transport systems have been shown to influence competitive fitness and biofilm-associated behaviors through integration with global regulatory networks. More directly, in *Klebsiella pneumoniae*, deletion of the *celB* gene similarly leads to significantly reduced biofilm formation and attenuated bacterial virulence [23], while in *Lactococcus lactis*, the PTS system containing *celB* is also involved in lactose transport [24]. These observations suggest that sugar-specific PTS components may function beyond substrate uptake, potentially influencing community structure and matrix-associated phenotypes. Although direct evidence linking *celB* to biofilm regulation in other organisms remains limited, the broader literature supports a conceptual framework in which carbohydrate transport systems are linked to virulence-related traits.

The precise mechanism by which erythritol modulates *celB* expression remains to be determined. One possibility is that erythritol perturbs carbohydrate-associated signaling within the PTS network, thereby influencing transcriptional responses that affect EPS-related genes. Because carbohydrate transport systems interface with global metabolic regulation in *S. mutans*, altered PTS activity may indirectly influence glucosyltransferase expression or glucan assembly processes. Alternatively, erythritol may induce broader metabolic adjustments that secondarily affect matrix synthesis. Further investigation will be required to clarify whether *celB* exerts direct regulatory effects on EPS-associated genes or functions as part of a larger metabolic signaling cascade.

Importantly, our results support a model in which erythritol may influence *S. mutans* virulence not solely through metabolic inertness but also through modulation of carbohydrate transport-associated pathways. This perspective expands the conceptual framework of anti-cariogenic polyols, suggesting that certain sugar substitutes may actively interfere with biofilm regulatory networks rather than merely serving as non-fermentable alternatives.

Notably, we further validated our findings under anaerobic conditions to better approximate the microenvironment of cariogenic biofilms. The inhibitory effects of erythritol on biofilm formation and EPS production, as well as the phenotypic changes observed in the Δ*celB* mutant, were consistent with those obtained under aerobic conditions. These results suggest that the regulatory role of *celB* and the anti-biofilm effects of erythritol are not dependent on oxygen availability.

Given that cariogenic biofilms are often characterized by low-oxygen or anaerobic niches, these findings strengthen the potential physiological relevance of our study and support the notion that erythritol-mediated modulation of carbohydrate transport systems may occur under conditions more reflective of the in vivo oral environment.

Several limitations should be acknowledged. The present study was conducted using a single-species static in vitro biofilm model, which does not fully recapitulate the complexity and dynamic conditions of oral multispecies biofilms. Second, although *celB* deletion reduced EPS production, the downstream regulatory mechanisms linking *celB* to glucosyltransferase expression or glucan assembly were not directly examined. It remains unclear whether *celB* represents a direct molecular target of erythritol or whether its downregulation reflects broader metabolic remodeling.

## 5. Conclusions

This study provides evidence that erythritol suppresses EPS production and biofilm formation in *S. mutans* and implicates *celB* as a contributing factor in this process. By linking erythritol exposure to modulation of a carbohydrate transport component associated with matrix synthesis, our work advances understanding of the molecular basis underlying erythritol’s anti-cariogenic activity while highlighting carbohydrate transport systems as potential targets for biofilm control.

## Data Availability

The original contributions presented in this study are included in the article/Supplementary Materials. Further inquiries can be directed to the corresponding authors.

## Supplementary Materials

The following supporting information can be downloaded at: https://www.mdpi.com/article/10.3390/microorganisms14040782/s1, Table S1: Primer sequences used for gene deletion and qPCR analysis. Table S2: Gene expression matrix of RNA-seq data. Normalized counts for all genes in *S. mutans* UA159 cultured with or without 10% erythritol (*n* = 3). The supporting information will be made available online.
